# Evaluating the Prophylactic and Nephroprotective Effects of Vitamin D and Metformin in Diabetic Nephropathy

**DOI:** 10.1155/omcl/5370323

**Published:** 2025-07-26

**Authors:** Lavanya B. Ramegowda, Prashant Vishwanath, Paramahans V. Salimath, Manjunath S. Shetty, Srinath K. Marulaiah, Shobha C. Ramachandra, Akila Prashant

**Affiliations:** ^1^Center of Excellence in Molecular Biology and Regenerative Medicine, Department of Biochemistry, JSS Medical College, JSS Academy of Higher Education and Research, Mysuru, Karnataka, India; ^2^Special Interest Group on Human Genetics and Rare Disorders, JSS Medical College, JSS Academy of Higher Education and Research, Mysuru, Karnataka, India; ^3^Department of Nephrology, JSS Medical College, JSS Academy of Higher Education and Research, Mysuru, Karnataka, India; ^4^Department of General Medicine, JSS Medical College, JSS Academy of Higher Education and Research, Mysuru, Karnataka, India

**Keywords:** antioxidant effects, diabetic nephropathy, metformin, oxidative stress, vitamin D

## Abstract

**Introduction:** Diabetic nephropathy (DN), a major complication of diabetes mellitus (DM) and a leading cause of end-stage renal disease (ESRD) globally, is characterized by oxidative stress (OS), chronic inflammation, and progressive fibrosis. Despite existing treatment options, disease progression remains a challenge. This study evaluates the therapeutic potential of vitamin D, alone and in combination with metformin, in mitigating DN progression in streptozotocin (STZ) induced diabetic rats.

**Methods:** Male Wister rats were induced with diabetes using a single intraperitoneal injection of STZ and randomized into seven groups. Treatment regimens included vitamin D (5000 or 8000 IU), metformin (250 mg), or a combination, administered over 12 or 21 weeks. Fasting blood glucose (FBG), lipid profiles, renal function markers, and OS indicators were assessed. Renal tissues were examined via histopathological analysis to assess structural changes, and immunohistochemistry (IHC) was performed to evaluate the expression of key proteins involved in inflammation (transforming growth factor–beta [TGF-β]), fibrosis (VEGF), and OS (nuclear factor erythroid 2-related factor 2 [Nrf2]), and vitamin D receptor (VDR) signaling.

**Results:** Vitamin D treatment caused a dose-dependent decrease in FBG, with the vitamin D and metformin combination therapy achieving the greatest decrease (−49.8%) by week 21. Triglyceride levels were significantly reduced (−50%), while HDL levels remained stable. Combination therapy significantly reduced hydrogen peroxide (H_2_O_2_) (−36.84%) and nitric oxide (NO) (−14.29%) and enhanced antioxidant enzyme activity: glutathione reductase (GR) (+250%), Superoxide dismutase (SOD) (+11.33%), and Glutathione peroxidase (GPx) (+62.83%). Histological analysis revealed preserved renal architecture and reduced fibrosis in treated groups, particularly in those receiving combination therapy. IHC showed increased VDR and Nrf2 expression, reduced VEGF and TGF-β levels, reflecting attenuation of inflammation, fibrosis, and oxidative damage.

**Conclusion:** Vitamin D, particularly in combination with metformin, significantly attenuates DN progression by enhancing metabolic control, reducing OS, and preserving renal function. These findings support its potential as an effective adjunctive therapy in DN management and provide a foundation for future clinical investigations.

## 1. Introduction

Diabetic nephropathy (DN) is a major microvascular complication of diabetes mellitus (DM), particularly prevalent in individuals with type 1 and type 2 diabetes [1]. Globally, DN is the leading cause of end-stage renal disease (ESRD), accounting for a substantial burden on healthcare systems [2]. The progression of DN is primarily driven by chronic hypergycemia, which triggers oxidative stress (OS), inflammation, and fibrosis, ultimately impairing renal function [3]. Key histopathological features include thickening of the glomerular basement membrane (GBM), podocyte loss, mesangial matrix expansion, and extracellular matrix (ECM) accumulation, contributing to glomerulosclerosis and tubulointerstitial fibrosis [4]. Despite advances in glycemic control and overall diabetes management, the rising prevalence of DN underscores the urgent need for novel therapeutic strategies to preserve renal function in diabetic individuals [5].

Vitamin D, traditionally recognized for its role in calcium and phosphate homeostasis [6], has gained attention for its potential renoprotective effects due to its anti-inflammatory, antioxidant, and antifibrotic properties [7]. Dysregulated vitamin D metabolism in diabetes is associated with a heightened risk of DN progression [8]. Its renoprotective actions are mediated through key molecular pathways, particularly nuclear factor erythroid 2-related factor 2 (Nrf2) and transforming growth factor–beta (TGF- β), which regulate OS responses and fibrogenesis in the kidney [9, 10]. Additionally, vitamin D improves insulin sensitivity and glycemic control, potentially mitigating the development of DN in individuals with DM [11].

Metformin, a first-line pharmacologic agent for type 2 DM (T2DM), also exhibits renoprotective effects improving insulin sensitivity, reducing hyperglycemia, and modulating OS [12]. The combination of vitamin D and metformin offers a potentially synergistic therapeutic strategy, targeting both metabolic and molecular pathways involved in DN. This study aims to explore the prophylactic and nephroprotective roles of vitamin D alone and in combination with metformin, with a focus on their ability to modulate OS and mitigate the progression of DN.

## 2. Materials and Methods

### 2.1. Materials

Streptozotocin (STZ, Cat# S0130), Vitamin D (Cat# C9756-5GM), Metformin (Cat# PHR1084-500MG), β-Nicotinamide adenine dinucleotide 2′-phosphate reduced (NADPH, Cat# N7505), were procured from sigma Aldrich, St. Louis, USA. BCA protein estimation kit (Cat# 23227), Bovine serum albumin (BSA, Cat# 23209) were procured from Thermo Fischer Scientific, Waltham, MA, United States. NAD(P)H: quinone oxidoreductase 1 (NQO1, Cat# PALP69Hu01), VEGF (Cat# MAA143Hu24), TGF-β (Cat# MAB949Hu24), vitamin D receptor (VDR, Cat# sc13133), Superoxide dismutase (SOD, Cat# PAB960Hu01), Nrf2 (Cat # PAL947Hu01) from cloud clone. AMP-activated protein kinase (AMPK, Cat# 2532S). β-actin (Cat# 4967), Mouse anti-rabbit IgG horseradish peroxidase (HRP) (Cat# sc-2357), Goat anti-rabbit IgG HRP (Cat# sc-1004), Goat anti-mouse IgG HRP (Cat# sc-2005) were from Santa Cruz Biotechnology, Inc, Dallas, TX, USA. High throughput SOD assay kit (Cat# 7501-500-K) and high throughput glutathione peroxidase (GPx) assay kit (Cat# 7512-100-K) from R&D systems. Ethylenediaminetetraacetic acid (EDTA, Cat# 40648), RIPA buffer 1× (100 mL) was prepared as detailed and stored at −20˚C. The composition of 1× RIPA buffer –20 mM Tris-HCl (pH 7.5), 150 mM NaCl, 1 mM Na_2_EDTA, 1 mM EGTA, 1% NP-40, 1% Sodium deoxycholate, 2.5 mM sodium pyrophosphate, 1 mM β-glycerophosphate, 1 mM Na_2_VO_4_, 1 g/mL leupeptin. Acrylamide (Cat# 22794); bis-acrylamide (Cat# 38516); coomassie blue G-250 (Cat# 64222); EDTA (Cat# 40648); glycine (Cat# 66327); polysorbate 20 (Tween 20) (Cat #23610); sodium lauryl sulfate AR (Cat# #54468); TEMED (Cat# 52145); trichloroacetic acid (Cat# 90544); tris buffer AR (#71033) were procured from SRL – Mumbai, Maharashtra, India. PVDF Membrane from Pall Corporation (Cat# BSP0161), Precision Plus Protein WesternC Blotting Standards (Cat# 1610376), StrepTactin-HRP (Cat# 1610380), Clarity Western enhanced chemiluminescent (ECL) (Cat# 1705061) were procured from Bio-Rad.

### 2.2. Animal Study

A total of 102 male Wistar rats, aged 7 weeks and weighing 160–180 g, were obtained from the Experimental Animal Centre, Department of Studies in Zoology, University of Mysore, Karnataka, India. The rats were housed in standard laboratory conditions, with a controlled temperature of 25°C, a 12-h light–dark cycle, and unrestricted access to a standard pellet diet (SPD) and water. All the experimental protocols were approved by Animal Ethical Committee of University of Mysore (Approval number UOM/IAEC/14/2022) and this study was conducted as per the Committee for the purpose of control and supervision of experiments on animals (CPCSEA) guidelines [13]. The study was carried out in compliance with the ARRIVE guidelines.

### 2.3. Induction of Diabetes

Following overnight fasting, the rats were intraperitoneally (i.p.) injected with a single dose of STZ at a dose of 45 mg/kg body weight (B.W.) to induce diabetes. Rats in the control SPD group received equivalent vehicle injections. Three days after the STZ injection, fasting blood glucose (FBG) levels were measured, and rats with FBG levels > 200 mg/dL were classified as diabetic and selected for subsequent experiments.

### 2.4. Experimental Design for Strategy 1: Prophylactic Impact of Vitamin D on the Prevention of DN

Following the induction of diabetes, diabetic rats were randomly divided into seven equal groups, except diabetic control group (*n* = 7 rats per group) to assess the prophylactic effects of vitamin D on the prevention of DN. Experimental groups were as follows: Group 1: Control; Group 2: Diabetic control; Group 3: DN control; Group 4: Diabetic group treated with vitamin D (5000 IU/kg B.W.) [14, 15]; Group 5: Diabetic group treated with vitamin D (8000 IU/kg B.W.) [16]; Group 6: Diabetic group treated with vitamin D and metformin (5000 IU/kg B.W. + 125 mg/kg B.W.) Group 7: Diabetic group treated with vitamin D and metformin (8000 IU/kg B.W. + 125 mg/kg B.W.); Group 8: Diabetic group treated with metformin (125 mg/kg B.W.). A detailed description of the treatment protocols is provided in Table S1. Metformin and Vitamin D were administered orally three times a week starting from the 3^rd^ week after the induction. Simultaneously, in Group 2 and 3 animals were administered sodium citrate buffer as a procedural control. FBG levels and B.W. were measured every 3 weeks, to monitor the progression of diabetes. Metformin, an antihyperglycemic drug, was used as a positive control. At the end of the 12^th^ week, Group 3 animals were placed in metabolic cages to collect 24 h urine samples for measuring microalbuminuria. The presence of microalbuminuria (> 30 mg/24 h) confirmed the development of DN in diabetic rats. By the 12^th^ week, all animals were euthanized by CO_2_ asphyxiation followed by cervical dislocation, as recommended by CPCSEA guidelines. Euthanization of rat was performed in a location separate from the room where the rats were housed to minimize anxiety, pain, and distress. Blood was collected via the retro-orbital puncture and along with urine samples for biochemical analysis. The serum samples were stored at −80°C for future biochemical estimations. Following blood collection, the rats were sacrificed by cervical dislocation, and their vital organs (kidney, liver, and pancreas) were collected for further analysis.

### 2.5. Experimental Design for Strategy 2: Nephroprotective Effects of Vitamin D in DN

In Strategy 2, the nephroprotective effects of vitamin D were evaluated in a therapeutic context, specifically after the onset of DN. DN was confirmed in the experimental model by the presence of microalbuminuria in diabetic rats by the 12^th^ week of the study. Following DN confirmation, the rats were randomized into six experimental groups, each groups receiving treatment protocols consistent with those used in Strategy 1. In this protocol, however, vitamin D administration began in the 13^th^ week and continued through until the 21^st^ week, at which point all animals underwent necropsy for histological and biochemical evaluation. This strategy differs from Strategy 1, where vitamin D was administered as a prophylactic intervention starting from an earlier time point before DN onset. In contrast, Strategy 2 aims to assess the therapeutic efficacy of vitamin D as a postdiagnosis intervention. By initiating treatment after DN has developed, this approach seeks to determine the potential of vitamin D to mitigate progression or reverse damage within the renal tissues in the context of established DN.

### 2.6. Preparation of Kidney Homogenates

Renal tissues from all rat groups were harvested, perfused with ice-cold saline, and homogenized in RIPA buffer using a probe sonicator (Vibracell-VCX500, Sonics, Sonics & Materials Inc, Newtown, Connecticut, USA). The homogenates were then centrifuged at 12,000 rpm for 15 min at 4°C and the supernatant was collected for subsequent assays of antioxidant enzymes, OS markers, and western blot analysis. Total protein levels were quantified using a commercially available kit from Thermo Scientific, with BSA used as the reference standard.

### 2.7. Assessment of Renal Dysfunction

The levels of serum FBG, lipid profile (total cholesterol, HDL, triglycerides), and renal function markers (urea, creatinine, uric acid, calcium, phosphorus, and BUN) were estimated using commercially available Roche kits, according to the manufacturer's protocol on a fully automatic Cobas c501 analyzer (Roche, USA). Serum vitamin D levels were determined using a Cobas 601 immunoassay analyzer (Roche, USA). Microalbuminuria (24-h urine) were quantified from the collected urine samples using a Cobas c501 chemistry analyzer (Roche, USA).

### 2.8. Renal OS Markers

Renal OS was assessed by measuring the levels of nitric oxide (NO) and hydrogen peroxide (H_2_O_2_) in kidney homogenates. NO levels were quantified through the Griess assay by measuring nitrite accumulation, a stable NO metabolite. In this assay, nitrite reacts with the Griess reagent to form a purple azo dye, with absorbance measured at 540 nm against a sodium nitrite standard curve [17]. H_2_O_2_ levels were assessed using the ferrous oxidation-xylenol orange (FOX1) assay. In this protocol, 10 μL of kidney homogenate, normalized to 20 μg of protein, was mixed with 190 μL of FOX1 reagent. The mixture was thoroughly mixed and incubated at room temperature (RT) for 30 min. After incubation, the absorbance was measured at 560 nm to determine the concentration of H_2_O_2_.

### 2.9. Renal Antioxidant Parameters

SOD activity in kidney homogenate samples was measured using the R&D Systems high throughput SOD assay kit. Protein concentrations were normalized to 50 µg, 25 µL of serially diluted standards, and samples were loaded into a 96-well plate. SOD activity was determined by measuring absorbance at 450 nm every minute for 10 min at RT using a multimode plate reader (Enspire 2300, PerkinElmer, Waltham, MA, USA). Results were calculated according to the manufacturer's protocol. GPx activity was measured in kidney homogenates samples using the R&D Systems high throughput GPx assay kit. A total of 20 µL of GPx standards and protein samples normalized to 50*µ*g were added to designated wells. GPx activity was determined by measuring absorbance at 340 nm every 30 s over 30 min. Results were calculated according to the manufacturer's protocol. Glutathione reductase (GR) activity in kidney homogenate was measured by monitoring the oxidation of NADPH. For the assay, 20 μL of the sample, normalized to 50 μg of protein, was added to 200 μL of assay buffer containing 1 mM glutathione disulfide (GSSG), 0.2 mM NADPH, and 0.1 M potassium phosphate buffer with 2 mM EDTA. After mixing the contents, the decrease in absorbance at 340 nm was monitored over 2 min, as the reduction of GSSG to Glutathione (GSH) consumes NADPH, resulting in a proportional decrease in absorbance, indicative of GR activity.

### 2.10. Western Blot

Protein expression in kidney homogenates was assessed by Western blotting. Samples were separated using 12% SDS-PAGE, with 25 μg of total protein loaded per lane. Proteins were transferred to polyvinylidene fluoride (PVDF) membrane, which was blocked with 5% nonfat skimmed milk in 1× Tris-buffered saline (TBS) for 2 h at RT. After blocking, the membrane was incubated overnight at 4°C with primary antibodies targeting VDR, NQO1, VEGF A, TGF-β, AMPK, SOD superoxide dismutase, and β-actin. Following primary antibody incubation, the membrane was washed three times with 1× TBS-Tween (TBST) for 10 min each. Secondary antibody incubation was performed for 2 h at RT on the gel rocker (Compact Digital Rocker, China). After washing, the membrane was developed using an ECL reagent (Biorad, USA). Protein bands were visualized using a chemiluminescent gel documentation system (Alliance Q9, UK) and band intensities were quantified with ImageJ software. Results were expressed as fold changes by normalizing the densitometric values of the test samples to β-actin (control).

### 2.11. Renal Histopathological Examination

Renal tissue samples were fixed in 10% formalin and dehydrated in a graded series of ethanol. The tissues were embedded in paraffin and sectioned into 4 μm slices. These sections were stained with hematoxylin and eosin (H&E), Periodic acid-Schiff (PAS), and Masson's trichrome stain (MTS) for histopathological examination under a light microscope. The histopathological evaluations were conducted in a blinded manner to minimize any observer bias.

### 2.12. Renal Immunohistochemistry (IHC)

Renal tissues were sectioned into 4 μm slices, deparaffinized, and permeabilized by incubation at 65°C in 0.1 M sodium citrate for 2 h. To block endogenous peroxidase activity, the sections were treated with 3% hydrogen peroxides for 15 min. Following this, the slides were incubated in a 100°C water bath for 10 min in 0.01M phosphate-buffered saline (PBS). Tissue sections were blocked with 2% BSA for 30 min. Primary antibodies targeting VDR, Nrf2, TGF-β, and VEGF were applied followed by overnight incubation at 4°C. After washing, the sections were incubated with HRP-conjugated secondary antibody at 37°C for 1 h. Visualization was performed using a 3,3′-diaminobenzidine (DAB) substrate for 4 min. Observations and images were captured under a light microscope. All IHC assessments were performed by who were blinded to the treatment groups to ensure unbiased results.

### 2.13. Statistical Analysis

Given the small sample size (*n* = 7 per group), normality was assessed using visual methods, including histograms and boxplots, and was further confirmed using the Shapiro–Wilk test. Values are represented as mean ± SE for normally distributed variables. Comparisons between multiple groups were performed using a one-way analysis of variance (ANOVA). When ANOVA indicated significant differences, Tukey's post hoc test was used for pairwise comparisons to identify specific group differences.

Western blot data were analyzed using densitometry, and protein expression differences between groups were compared using one-way ANOVA followed by Tukey's post hoc test for pairwise comparisons. For histopathological and IHC analysis, a semiquantitative scoring system was applied to assess the degree of renal damage, and comparisons between groups were performed using the chi-square test or Fisher's exact test for categorical data.

All statistical analyses were conducted using Graph Pad Prism 9.0 (Graph Pad Software, La Jolla, CA, USA) or SPSS version 25 (IBM Corp., Armonk, NY, USA). A *p* − value  < 0.05 was considered statistically significant, with Bonferroni correction applied for multiple comparisons where necessary.

## 3. Results

### 3.1. Impact on B.W.

**Figure 1:**
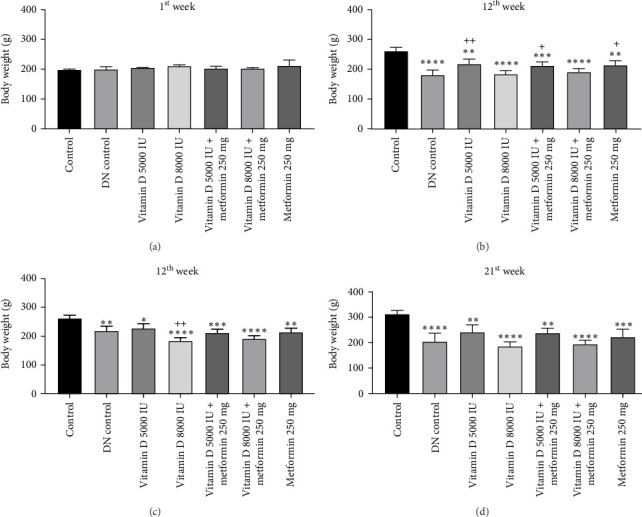
(a) and (b) Effect of vitamin D and metformin on body weight of STZ-induced diabetic rats (12-week experiment). (c and d) Effect of vitamin D and metformin on body weight of STZ induced DN rats (21-week experiment). Values are represented as mean ± SE. One-way ANOVA followed by Tukey's post hoc test was used for multiple group comparisons. Superscripts *⁣*^*∗*^, *⁣*^*∗∗*^, *⁣*^*∗∗∗*^, and *⁣*^*∗∗∗∗*^ indicate significance at *p*-value < 0.05, < 0.01, < 0.001, and 0.0001, respectively, with all groups compared to the control group. Superscripts ^+^, ^++^, ^+++^, and ^++++^ indicate significance at *p*-value < 0.05, < 0.01, < 0.001, and < 0.0001, respectively, with all groups compared to DN control group.

During the 12-week experiment, STZ-induced diabetic rats exhibited significant weight loss compared to a control group. By the end of the 12^th^ week, the B.W. of rats treated with vitamin D, either alone or in combination with metformin, was maintained at a higher level compared to the DN control group (Figure 1a,b). Similarly, in the 21-week experiment, STZ-induced DN rats exhibited significant weight loss compared to normal rats. However, by the end of the 21^st^ week, the B.W. of rats treated with Vitamin D, either alone or in combination with metformin, showed a significant increase compared to the DN control group. This indicated that vitamin D and metformin have a beneficial effect on B.W. maintenance in the context of DN (Figure 1c, d). A detailed week by week B.W. profile for all groups is provided in the Supporting Information (Figure S1a, b).

### 3.2. FBG Levels

**Figure 2:**
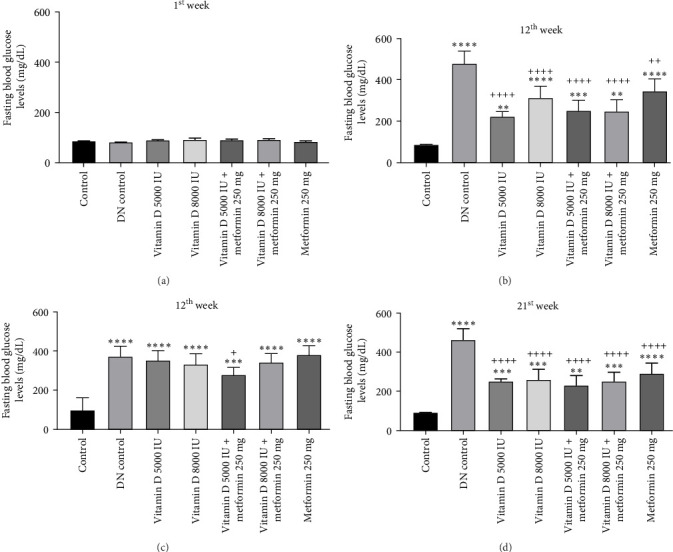
(a) and (b) Effect of vitamin D and metformin on FBG levels in STZ-induced diabetic rats (12-week experiment). (c and d) Effect of vitamin D and metformin on FBG levels in STZ-induced DN rats (21-week experiment). Values are represented as mean ± SE. One-way ANOVA followed by Tukey's post hoc test was used for multiple group comparisons. Superscripts *⁣*^*∗*^, *⁣*^*∗∗*^, *⁣*^*∗∗∗*^, and *⁣*^*∗∗∗∗*^ indicate significance at *p*-value < 0.05, < 0.01, < 0.001, and 0.0001, respectively, with all groups compared to the control group. Superscripts ^+^, ^++^, ^+++^, and ^++++^ indicate significance at *p*-value < 0.05, < 0.01, < 0.001, and < 0.0001, respectively, with all groups compared to DN control group.

The administration of STZ resulted in a significant increase in FBG levels in DN control groups compared to the control group throughout the 12-week study. However, by the end of the 12^th^ week, STZ-induced diabetic rats treated with 5000 and 8000 IU of vitamin D showed a decrease (− 49.8%) in FBG levels, while the other groups exhibited a nonsignificant reduction (Figure 2a, b). Similarly, in the 21-week study, STZ-induced DN rats displayed significantly elevated FBG levels compared to the control group. By the 21^st^ week, rats treated with 8000 and 5000 IU of vitamin D, either alone or in combination with metformin, showed a significant decrease (− 50%) in FBG levels compared to the DN control group (Figure 2c,d). A detailed week by week FBS profile for all groups is provided in Figure S2a, b.

### 3.3. Assessment of Microalbuminuria

**Figure 3:**
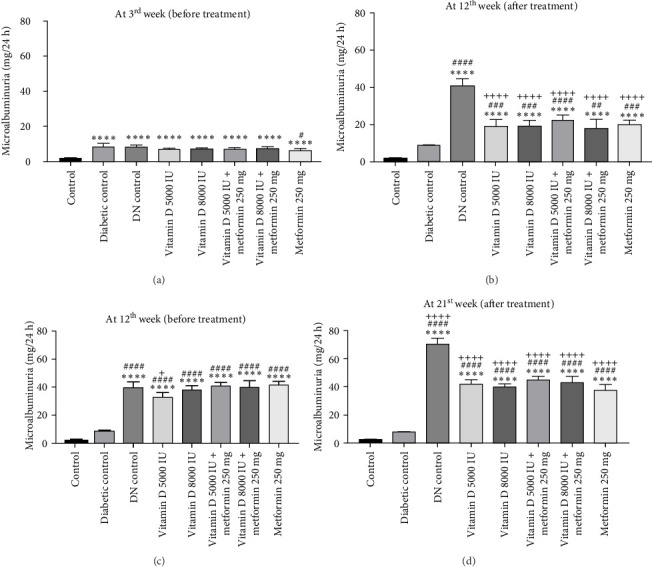
(a) and (b) Effect of vitamin D and metformin on microalbuminuria in STZ-induced diabetic rats at 3^rd^ week and 12^th^ weeks (12-week experiment). (c and d) Effect of vitamin D and metformin on microalbuminuria in STZ-induced DN rats at 12^th^ week and 21^st^ week (21-week experiment). Values are represented as mean ± SE. One-way ANOVA followed by Tukey's post hoc test was used for multiple group comparisons. Superscripts *⁣*^*∗*^, *⁣*^*∗∗*^, *⁣*^*∗∗∗*^, and *⁣*^*∗∗∗∗*^ indicate significance at *p*-value < 0.05, < 0.01, < 0.001, and 0.0001, respectively, with all groups compared to the control group. Superscripts ^#^, ^##^, ^###^, and ^####^ indicate significance at *p*-value < 0.05, < 0.01, < 0.001, and < 0.0001, respectively, with all groups compared to diabetic control group. Superscripts ^+^, ^++^, ^+++^, and ^++++^ indicate significance at *p*-value < 0.05, < 0.01, < 0.001, and < 0.0001, respectively, with all groups compared to DN control group.

During the 12-week experiment, 24-h urine samples were collected from all experimental groups in the 3^rd^ week, before initiating treatment with vitamin D or the combination of vitamin D and metformin. Microalbuminuria levels key marker for DN were measured at the 12^th^ week to assess disease progression, By this point, rats in the DN control group had progressed to nephropathy stage, with microalbuminuria levels reaching 30 mg/L, indicating the onset of renal damage. After confirming the development of nephropathy, the treatment was stopped. However, the remaining groups treated with vitamin D, either alone or in combination with metformin, did not reach the DN stage, demonstrating the protective effects of these treatments against the progression of kidney damage (Figure 3a, b).

In the 21-week experiment, 24-h urine samples were collected from all experimental groups in the 12^th^ week to confirm microalbuminuria levels, which served as an indicator of kidney damage in the DN control group. Following this confirmation, treatment with vitamin D and the combination of vitamin D and metformin was initiated. By the end of the 21^st^ week, microalbuminuria levels were effectively stabilized in both the vitamin D and the vitamin D with metformin treatment groups, showing marked improvement compared to the DN control group. This suggests that these treatments mitigated further kidney damage and helped maintain renal function, highlighting their potential therapeutic benefit in managing DN (Figure 3c, d).

### 3.4. Serum Profiles

This study demonstrates significant alterations in lipid profiles in diabetic and DN control groups. Cholesterol levels were elevated in the diabetic group compared to controls but decreased in the DN control group. Vitamin D supplementation, at both 5000 and 8000 IU, resulted in a dose-dependent reduction in cholesterol levels, with the most pronounced effect observed in the 8000 IU vitamin D combined with the 250 mg metformin group. Triglyceride levels, which were elevated in the diabetic control group, were significantly reduced (− 50%) following treatment with 8000 IU vitamin D, both alone and in combination with metformin. Notably, no significant changes were observed in HDL levels across all groups. Overall, these findings suggest that vitamin D, particularly at 8000 IU and when combined with metformin, exerts beneficial effects on lipid metabolism in diabetic and DN models, potentially enhancing nephroprotective outcomes by improving the lipid profile. This may play a key role in mitigating cardiovascular risk factors associated with DN (Supporting Information; Table S2 and Table S3; Lipid profile).

In the 12^th^ week of the study, phosphorus levels decreased in the diabetic group compared to controls but increased in the DN control group. However, treatment with vitamin D 5000 IU combined with 250 mg metformin significantly reduced phosphorus levels compared to both control and DN control groups. By the 21^st^ week, phosphorus levels had decreased in all treatment groups except for the metformin-treated group, where phosphorus levels remained similar to controls. Despite these alterations in phosphorus, no significant changes were observed in calcium, urea, uric acid, creatinine, or BUN levels across any of the groups. Uric acid levels, however, significantly decreased in all treated groups, while BUN levels showed a slight increase across all groups, except for the 8000 IU vitamin D combined with 250 mg metformin group, where BUN levels remained comparable to the control, diabetic, and DN control groups. The stability of calcium, urea, and BUN levels suggests that renal function and overall metabolic status were maintained throughout the study, explaining the lack of significant differences in these parameters. Metformin's ability to maintain phosphorus levels close to control values may be related to its influence on glucose and lipid metabolism, which could indirectly stabilize phosphate handling in the kidneys. Additionally, metformin's role in modulating phosphate transport and inhibiting phosphate reabsorption in renal cells, explains its influence on phosphorous homeostasis without disrupting overall renal or metabolic function (Supporting Information; Table S2 and Table S3; renal function markers).

In diabetic conditions, serum vitamin D levels progressively declined compared to Controls. This reduction in vitamin D levels correlated with the exacerbation of DN. Upon administration of vitamin D supplementation, there was a noticeable increase in serum vitamin D levels compared to the control group. This supplementation not only normalizes vitamin D status but also potentially mitigates the progression of DN by modulating the associated pathophysiological mechanisms (Supporting Information; Table S2; and Table S3; serum vitamin D levels).

### 3.5. OS Markers

Our study demonstrates that OS, characterized by elevated H_2_O_2_ levels, and plays a key role in the pathogenesis of diabetes and DN. In kidney homogenates, H_2_O_2_ levels were significantly increased in both diabetic and DN control groups compared to controls, reflecting heightened OS. However, treatment with vitamin D (5000 and 8000 IU) combined with metformin 250 mg, as well as metformin alone, effectively reduced (− 36.84%) H_2_O_2_ levels, with the most notable normalization observed in the group treated with vitamin D 8000 IU and metformin. This suggests that these interventions possess antioxidant properties capable of mitigating OS in diabetic and DN conditions (Supporting Information; Table S2; Renal function markers). Similarly, NO levels were significantly elevated in both diabetic and DN control groups compared to controls, indicating dysregulated NO metabolism. While all treatment groups exhibited increased NO levels compared to controls, the combination of vitamin D 8000 IU with metformin, and metformin alone significantly reduced (−14.29%) NO levels in a dose-dependent manner, bringing them closer to normal levels. These findings indicate that the combination of vitamin D and metformin exerts a dual modulatory effect by reducing both H_2_O_2_ and NO levels, thereby offering protection against oxidative damage in DN (Supporting Information; Table S3; Renal function markers).

### 3.6. Antioxidant Defense Mechanism

The antioxidant defense mechanisms, involving key enzymes, such as GR, SOD, and GPx were significantly compromised in both diabetes and DN control groups, as evidenced by a significant reduction in their activity. However, treatment with vitamin D and metformin significantly restored and enhanced the activity of these enzymes across all treated groups, with increases of GR (+ 250%), SOD (+ 11.33%), and GPx (+ 62.83%). These findings suggest an improved cellular antioxidant response, contributing to the protective effects observed against OS and DN (Supporting data; Table S2 and Table S3 Antioxidant defense).

### 3.7. Modulation of Angiogenesis, Fibrosis, and OS by Vitamin D and Metformin in DN

**Figure 4:**
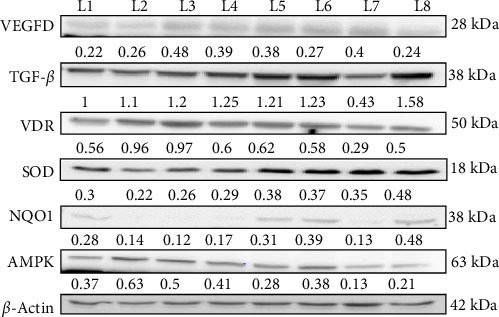
Depicts the prophylactic effect of vitamin D and metformin on protein levels in STZ-induced diabetic rats. Western blot data were analyzed through densitometry, with protein expression differences between groups assessed using one-way ANOVA followed by Tukey's post hoc test for pairwise comparisons. Lanes labeled as L1- Control; L2- Diabetic control; L3- DN control; L4- Vitamin D 5000 IU; L5- Vitamin D 8000 IU; L6- Vitamin D 5000 IU + Metformin 250 mg; L7- Vitamin D 8000 IU + Metformin 250 mg; L8- Metformin 250 mg.

**Figure 5:**
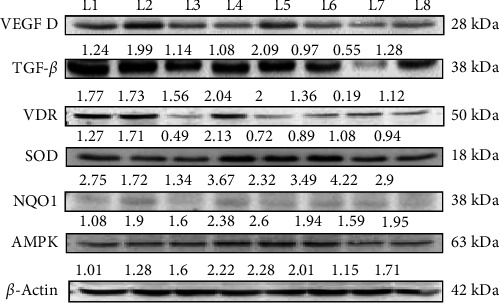
Illustrates the nephroprotective effect of vitamin D and metformin on protein levels in STZ-induced DN rats. Western blot data analysis and statistical testing were performed similarly to the prophylactic group. Lanes labeled as L1- Control; L2- Diabetic control; L3- DN control; L4- Vitamin D 5000 IU; L5- Vitamin D 8000 IU; L6- Vitamin D 5000 IU + Metformin 250 mg; L7- Vitamin D 8000 IU + Metformin 250 mg; L8- Metformin 250 mg.

In this western blot study investigating the prophylactic (Figure 4) and nephroprotective effects (Figure 5) of vitamin D on DN, we examined how vitamin D (5000 and 8000 IU) alone and in combination with metformin influenced key protein markers in an STZ-induced diabetic and DN rat model. In DN control rats, VEGF dimer expression was elevated, indicative of abnormal angiogenesis associated with DN. Treatment with the combination of vitamin D 5000 IU and metformin reduced VEGF levels, suggesting a therapeutic reduction in aberrant angiogenic activity. However, treatment with vitamin D at 8000 IU combined with metformin led to an increase in VEGF expression, likely due to dose-dependent feedback effect on angiogenic pathways. This differential response highlights the nuanced effect of vitamin D dosage on angiogenesis in DN. TGF-β, a maker associated with fibrosis, was elevated across all diabetic and DN control groups. However, significant reductions were observed in the group receiving 8000 IU vitamin D with metformin, indicating an antifibrotic benefit at higher vitamin D doses in combination with metformin. This finding indicates a potential therapeutic advantage of the higher dose for mitigating fibrotic progression in DN. In the prophylactic approach, VDR expression was upregulated in response to vitamin D supplementation, likely as a compensatory mechanism to diabetic rats. However, in the nephroprotective strategy, VDR levels were lower in DN control group compared to treated groups. Treatment with vitamin D and metformin improved VDR expression, suggesting that this combination might enhance receptor activity and vitamin D pathway engagement in the nephroprotective context. Antioxidant markers SOD and NQO1, were initially low in untreated diabetic rats, indicating impaired oxidative defense mechanisms. Treatment with vitamin D and metformin led to increased expression of these antioxidant markers, reflecting an enhancement in oxidative defense and potential protective effects against oxidative damage in DN. AMPK, a marker of metabolic stress, was elevated in the untreated diabetic controls, consistent with increased metabolic strain. The combination of vitamin D and metformin, especially at 8000 IU dose, was associated with reduced AMPK expression, suggesting an alleviation of metabolic stress under treatment. These findings highlight the differential effects of vitamin D in both prophylactic and nephroprotective contexts, with a notable synergy observed when combined with metformin. The results suggest that vitamin D, especially in combination with metformin, may offer protection against DN progression through modulation of key pathways involved in angiogenesis, fibrosis, OS, and metabolic regulation.

### 3.8. Renal Histopathological Evaluation of the Effects of Vitamin D and Metformin

**Figure 6:**
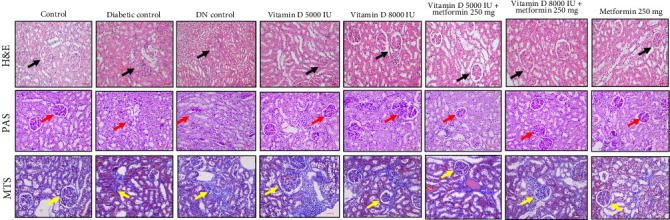
Prophylactic effect of vitamin D and metformin on histopathological changes in STZ-induced diabetic rats. Histopathological changes were assessed using hematoxylin and eosin (H&E) staining to observe alterations in glomerular and tubular morphology (black arrows), PAS staining highlighted glomerular basement membrane thickening and mesangial matrix expansion (red arrows), and Masson's trichrome stain (MTS) to visualize collagen deposition and assess the extent of fibrosis (yellow arrows) across all groups. Representative images from different renal tissue sections of each group were captured at an original magnification of 200×.

Histological and staining analyses revealed distinct renal morphological changes across the experimental groups. The control group exhibited normal kidney morphology, while the diabetic control showed moderate tubular degeneration and fibrosis. The DN control group displayed severe fibrosis, inflammation, and marked nephropathic changes. Treatment with 5000 IU of vitamin D resulted in mild tubular degeneration, but overall normal glomerular structure was maintained. In contrast, 8000 IU of vitamin D led to moderate tubular changes and mild cystic dilation. The combination of vitamin D and metformin demonstrated a protective effect, with several samples maintaining normal renal morphology and others exhibiting only mild inflammation. Treatment with metformin alone resulted in moderate tubular degeneration. PAS staining revealed improved glycogen accumulation and reduced PAS-positive staining in the treated groups. The combination of vitamin D and metformin notably reduced fibrosis compared to the diabetic and DN control groups, as shown in Figure 6. Overall, the combination therapy of vitamin D and metformin, particularly at higher doses effectively mitigated renal damage and fibrosis.

**Figure 7:**
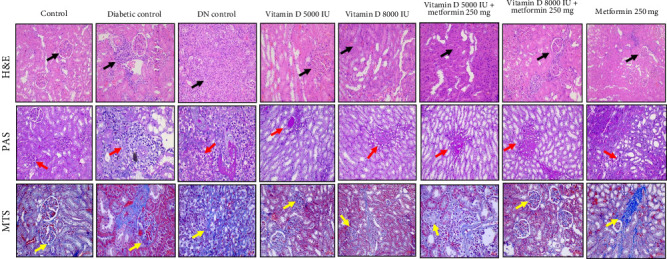
Nephroprotective effect of vitamin D and metformin on histopathological changes in STZ-induced DN rats. Histopathological changes were assessed using hematoxylin and eosin (H&E) staining to observe alterations in glomerular and tubular morphology (black arrows), PAS staining highlighted glomerular basement membrane thickening and mesangial matrix expansion (red arrows), and Masson's trichrome stain (MTS) to visualize collagen deposition and assess the extent of fibrosis (yellow arrows) across all groups. Representative images from different renal tissue sections of each group were captured at an original magnification of 200×.

Histological analyses showed significant variations in renal morphology and pathology among the groups. While the control group exhibited normal kidney architecture, both diabetic and DN control groups displayed severe inflammation, tubular degeneration, and nephritis, which are indicative of advanced nephropathy. Treatment with vitamin D, particularly when combined with metformin, demonstrated promising protective effects in mitigating renal damage and promoting regeneration. These treatments maintained or restored normal glomerular and tubular structures, and also reduced pathological changes, such as fibrosis and sclerosis, as shown in Figure 7. Supplementation with vitamin D and metformin was particularly effective in attenuating these pathological alterations, suggesting their potential therapeutic benefits in ameliorating diabetic kidney disease by preserving renal function and reducing structural damage.

### 3.9. Prophylactic Effects on Molecular Targets in DN

**Figure 8:**
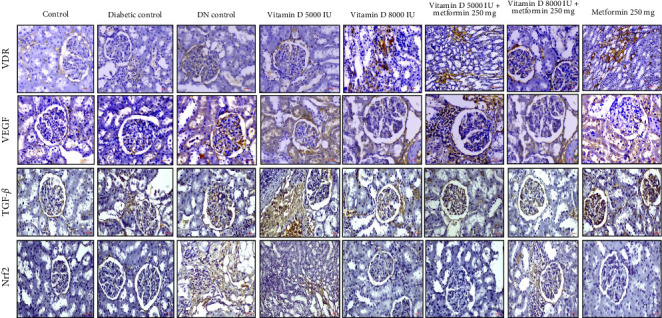
Prophylactic effect of vitamin D and metformin on immunohistochemical changes in STZ-induced diabetic rats. This analysis was performed to assess the expression of VDR, VEGF, TGF-β, and Nrf2 in renal tissue sections from all experimental groups. The representative images from different renal tissue sections are shown. Original magnification, 400×.

Vitamin D supplementation, both alone and in combination with metformin, demonstrated significant modulatory effects on key molecular targets associated with DN shown in Figure 8. Treatment with 5000 IU of vitamin D resulted in mild VDR expression, while 8000 IU of vitamin D led to moderate VDR expression in damaged renal tubules. The combination of vitamin D with metformin further enhanced VDR expression, suggesting a synergistic effect that could support renal defense mechanisms against DN development. In terms of VEGF expression, vitamin D 5000 IU combined with metformin showed moderate VEGF expression in regions of inflammation, whereas vitamin D 8000 IU with metformin exhibited a mild VEGF expression profile, suggesting that early intervention with this combination might temper pathological angiogenesis more effectively. Notably, TGF-β expression was significantly reduced in the groups treated with 8000 IU of vitamin D, both alone and in combination with metformin, indicating a reduction in inflammation and fibrosis compared to the DN control group. Additionally, Nrf2 expression, which was absent in the control group, was significantly upregulated in all treated groups, indicating an enhanced antioxidant response that may fortify renal tissue against OS in DN.

### 3.10. Therapeutics Effects on Molecular Targets in DN

**Figure 9:**
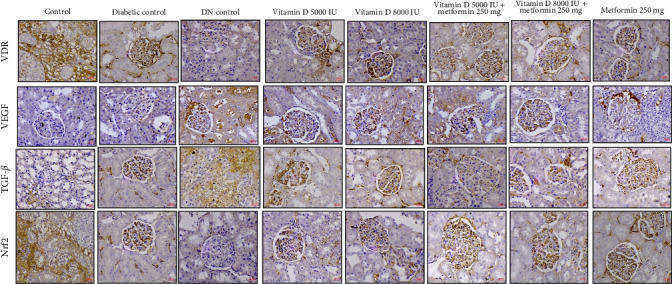
Nephroprotective effect of vitamin D and metformin on immunohistochemical changes in STZ-induced DN rats. This analysis was performed to assess the expression of VDR, VEGF, TGF-β, and Nrf2 in renal tissue sections from all experimental groups. The representative images from different renal tissue sections are shown. Original magnification, 400×.

The IHC reports highlight the dynamic effects of various treatments on key molecular targets implicated in DN. VDR expression, which is significantly diminished in diabetic conditions and absent in DN, was partially restored following treatment with vitamin D, particularly in combination with metformin. This restoration suggests that vitamin D and metformin may enhance receptor activity in damaged kidneys, promoting renal resilience post- DN diagnosis. Additionally, VEGF expression, which is elevated in diabetic conditions, was modulated by vitamin D and metformin treatments in a dose-dependent manner. This suggests that vitamin D and metformin may exert a dose-dependent modulation of VEGF, potentially aiding in the control of pathological angiogenesis in established DN. Furthermore, TGF-β1, a cytokine elevated in DKD and linked to fibrosis and tissue remodeling, was notably downregulated by the combined vitamin D and metformin therapy, indicating a therapeutic benefit in mitigating fibrosis and chronic inflammation. Lastly, the significant modulation of Nrf2 expression in the treated groups suggests that these interventions enhance the kidney's antioxidant defense mechanisms and shield against oxidative damage, as shown in Figure 9.

These findings underscore that vitamin D and metformin have distinct roles in both prophylactic and therapeutic settings. By modulating key molecular markers like VDR, VEGF, TGF-β1, and Nrf2 these treatments provide potential benefits in slowing DN progression and improving renal function, whether used to prevent or treat established DN.

## 4. Discussion

In this study, microalbuminuria served as a critical early marker for assessing the onset of DN in the experimental rat model, signaling initial glomerular damage induced by diabetes. Persistent microalbuminuria, a recognized predictor of DN progression, often precedes more severe proteinuria and advanced kideney impairment, highlighting its role as both a diagnostic and prognostic indicator. By using microalbuminuria as a criterion for DN occurrence, the study allowed for targeted intervention, with treatments initiated either prophylactically or post- DN onset to evaluate both preventive and therapeutic outcomes. The administration of STZ led to a significant rise in FBG levels in both diabetic and DN control groups over the 12- and 21-week periods. By the 12^th^ week, rats treated with 5000 IU of vitamin D showed a notable reduction in FBG, while other treatment groups did not show significant changes. By the 21^st^ week, treatment with 8000 IU of vitamin D, either alone or in combination with metformin, significantly lowered FBG levels compared to DN control groups. These results suggest that vitamin D exerts a dose-dependent effect on improving glycemic control in STZ-induced diabetic models, potentially through its known role in enhancing insulin sensitivity [18] and regulating glucose metabolism [19]. The additional glycemic improvements observed with the combination of metformin are likely due to its mechanisms of action, which include reducing hepatic glucose production and improving peripheral glucose uptake [20]. Interestingly, cholesterol levels did not change significantly across groups, suggesting that neither vitamin D nor metformin substantially modulated cholesterol metabolism in DN. However, triglyceride levels were significantly reduced in the groups treated with 5000 and 8000 IU of vitamin D, both alone and in combination with metformin. This reduction in TGs is attributable to vitamin D's effects on improving insulin sensitivity and modulating lipid metabolism [21–25]. Vitamin D is known to influence the expression of genes involved in fatty acid oxidation and TG synthesis, thereby reducing serum TG levels. The ability of metformin to enhance insulin sensitivity and inhibit hepatic gluconeogenesis [26], likely contributes further to the reduction in TG levels by promoting better lipid utilization and decreasing TG synthesis. The stable HDL levels across all groups indicate that neither vitamin D nor metformin had a significant impact on HDL metabolism or reverse cholesterol transport [27]. Overall, these findings highlight the potential of vitamin D and metformin in improving lipid profiles by targeting specific pathways in lipid metabolism in DN. Our findings are consistent with previous studies that demonstrate the glycemic and lipid-modulating effects of vitamin D and metformin in diabetic models [28–31].

The significant reduction in phosphorus levels observed in the DN control and all vitamin D treated groups, including those receiving vitamin D combined with metformin, compared to the diabetic control group, suggests a modulatory effect of vitamin D on renal phosphorus handling. Vitamin D plays a crucial role in phosphorus metabolism by enhancing intestinal absorption and regulating parathyroid hormone (PTH) levels, which influences renal phosphorus reabsorption. In DN, impaired kidney function disrupts mineral metabolism [32], but vitamin D supplementation, particularly at higher doses (5000 and 8000 IU), helps regulate PTH and promote phosphorous excretion, thereby reducing serum phosphorus levels. Metformin acts synergistically with vitamin D by improving metabolic control and reducing hyperphosphatemia, a common complication in DN. The absence of significant changes in calcium, urea, uric acid, creatinine, and BUN levels across all groups suggests that the interventions did not adversely affect renal filtration or excretion functions. Calcium homeostasis, which is tightly regulated by vitamin D, PTH, and calcitonin [33], remained stable, indicating that the doses of vitamin D were within a physiological range that supports normal calcium metabolism without inducing hypercalcemia. The stability of urea and creatinine, which are standard markers of GFR, suggest that renal excretory function was preserved in all groups, including those with DN and vitamin D treatment. This finding implies that vitamin D supplementation, even at 8000IU or in combination with metformin, did not exacerbate renal impairment. Additionally, unchanged uric acid levels indicate that vitamin D did not significantly influence purine metabolism or urate excretion [34] in this model. Overall, these findings highlight the potential of vitamin D as a safe therapeutic agent in managing DN without causing detrimental effects on key renal function markers.

Our study also reveals that under diabetic condition, a decline in serum vitamin D levels is closely linked to the progression of DN, driven by increased OS, inflammation, and fibrosis [35–37]. Vitamin D deficiency compromises key antioxidant defenses, as reflected by reduced activity of enzymes, such as GR, SOD, GPx, alongside elevated levels of H_2_O_2_ and NO levels. Vitamin D supplementation, particularly when combined with metformin, effectively restored serum vitamin D levels, reduced H_2_O_2_ and NO levels, and enhanced the activity of antioxidant enzymes [38, 39]. The restoration of antioxidant enzyme activities in treated groups underscores the ability of vitamin D and metformin to enhance the kidney's defense against OS. By reducing oxidative damage, these treatments slow the progression of DN and preserve renal tissue integrity. The simultaneous reduction in H_2_O_2_ and NO levels further indicates an overall improvement in oxidative balance.

Histological and staining analyses further highlighted the protective effects of vitamin D and metformin, particularly in combination, against renal damage associated with DN. The treatment with vitamin D, especially at higher doses, effectively preserved normal glomerular and tubular architecture while reducing pathological changes, such as tubular degeneration and fibrosis. These protective mechanism are likely mediated through the upregulation of VDR expression, which regulates anti-inflammatory and antifibrotic pathways [40, 41]. Our findings indicate that higher doses of vitamin D, particularly when administered alongside metformin, significantly enhance VDR activity, leading to improved anti-inflammatory responses that mitigate renal injury. Moreover, the combination therapy demonstrated a notable reduction in VEGF levels, which are often elevated in diabetic conditions. VEGF modulation suggests improved control of angiogenesis [42], which in turn reduces glomerular damage commonly seen in DN. Additionally, the significant suppression of TGF-β, a key mediator of renal fibrosis, indicates that the treatments effectively inhibit ECM accumulation and prevent glomerulosclerosis [43]. The activation of Nrf2 by vitamin D and metformin further enhances the kidney's defense against OS by upregulating antioxidant enzymes [44–48]. This multifaceted therapeutic approach, targeting key pathways, such as VDR for anti-inflammatory effects, VEGF for angiogenesis control, TGF-β for antifibrotic action, and Nrf2 for antioxidant defense, underscores the potential of vitamin D and metformin as promising therapies for mitigating the progression of DN and preserving renal function. This study is among the first to evaluate the dose-dependent effects of high dose vitamin D in combination with metformin on key molecular pathways associated with DN, such as VDR, VEGF, TGF-β and Nrf2. While previous studies have explored the individual effects of these agents on glycemic and lipid control, our findings provide new insights into their synergistic potential for reducing renal fibrosis, modulating angiogenesis, and enhancing antioxidant defenses.

Overall, our results support the hypothesis that combined vitamin D and metformin treatments offers a promising strategy for improving renal outcomes in patients with DN. These findings hold significant clinical relevance, as vitamin D deficiency is prevalent among diabetic patients and often correlates with poor renal outcomes. Our results suggest that vitamin D supplementation, particularly when combined with metformin, offer a targeted therapeutic approach to mitigating the progression of DN by addressing not only glycemic control but also OS and inflammation. This dual therapy could be particularly beneficial for patients at risk of rapid DN progression, making it a potential adjunct to current treatment protocols.

## 5. Limitations and Future Directions

While this study provides valuable insights into the therapeutic potential of vitamin D and metformin in DN, several limitations should be acknowledged. First, although the STZ-induced diabetic rat model effectively mimics several pathological features of human DN, it does not fully replicate the complexity and chronicity of the disease observed in human populations. Thus, extrapolation of findings should be made cautiously. Second, the doses of vitamin D (5000 and 8000 IU) and metformin (250 mg/kg) used in this preclinical study, although pharmacologically effective in rats, may not directly correspond to clinically equivalent or safe doses in humans. Dose optimization and pharmacokinetic comparisons are needed to ensure clinical relevance. Third, the study duration (12–21 weeks) may not adequately capture long term outcomes, such as progressive renal fibrosis or potential adverse effects. Furthermore, interindividual variability, coexisting comorbidities, and polypharmacy common in human diabetic populations were not accounted for in the model. Lastly, while the observed molecular changes in OS, inflammation, and fibrosis pathways are promising, further mechanistic validation in human renal cells or patient derived tissue samples is essential to confirm translational applicability.

## 6. Conclusions

Our study highlights the therapeutic potential of vitamin D, particularly in combination with metformin, in a preclinical model of DN. High dose vitamin D supplementation (8000 IU) significantly improved lipid profiles, attenuated OS, and preserved renal function. The combination therapy demonstrated the most pronounced effects, including reduced tubular degeneration, fibrosis, along with enhanced antioxidant enzyme activity. Furthermore, it modulated key molecular pathways by upregulating VDR and Nrf2 expression and downregulating proinflammatory and profibrotic markers, such as VFGF and TGF-β. While these findings provide compelling evidence for the renoprotective effects of vitamin D and metformin in an experimental setting, further clinical studies are warranted to validate their translational relevance and therapeutic applicability in human DN.

## Data Availability

The data that support the findings of this study are available from the corresponding author upon reasonable request.

## Supporting Information

Supporting Information Table S1. Treatment timeline for Strategy 1 and Strategy 2. Table S2. Effect of vitamin D and Metformin on serum markers in STZ induced diabetic rats. Table S3. Effect of vitamin D and Metformin on serum markers in STZ-induced DN rats. Both Table 2 and Table 3 include data on lipid profile, renal function markers, vitamin D levels, oxidative stress markers and antioxidant defense markers. Figure S1a. Effect of vitamin D and metformin on body weight of STZ-induced diabetic rats during the 12-week experimental period (Strategy 1). Figure S1b. Effect of vitamin D and metformin on body weight of STZ induced DN rats during the 21-week experimental period (Strategy 2). Figure S2a. Effect of vitamin D and metformin on FBG levels in STZ-induced diabetic rats during the 12-week experimental period (Strategy 1). Figure S2b. Effect of vitamin D and metformin on FBG levels in STZ-induced DN rats during the 21-week experimental period (Strategy 2).
