# Supplementary material for: Evaluating the Prophylactic and Nephroprotective Effects of Vitamin D and Metformin in Diabetic Nephropathy
Source: Oxid Med Cell Longev. 2025 Jul 26;2025:5370323. doi: 10.1155/omcl/5370323 (PMC12317812; doi:10.1155/omcl/5370323)
Supplement: Supporting Information — Table S1. Treatment timeline for Strategy 1 and Strategy 2. Table S2. Effect of vitamin D and Metformin on serum markers in STZ induced diabetic rats. Table S3. Effect of vitamin D and Metformin on serum markers in STZ-induced DN rats. Both Table 2 and Table 3 include data on lipid profile, renal function markers, vitamin D levels, oxidative stress markers and antioxidant defense markers. Figure S1a. Effect of vitamin D and metformin on body weight of STZ-induced diabetic rats during the 12-week experimental period (Strategy 1). Figure S1b. Effect of vitamin D and metformin on body weight of STZ induced DN rats during the 21-week experimental period (Strategy 2). Figure S2a. Effect of vitamin D and metformin on FBG levels in STZ-induced diabetic rats during the 12-week experimental period (Strategy 1). Figure S2b. Effect of vitamin D and metformin on FBG levels in STZ-induced DN rats during the 21-week experimental period (Strategy 2). [file 5370323.f1.docx]

**Title: Evaluating the Prophylactic and Nephroprotective Effects of Vitamin D and Metformin in Diabetic Nephropathy**

**Supplementary Data**:

Table S1: Treatment timeline for Strategy 1 and Strategy 2.

| Sl. No. | Groups | Treatment | Strategy 1 | | Strategy 2 | |
| --- | --- | --- | --- | --- | --- | --- |
|  |  |  | Treatment Start (Week) | Treatment End (Week) | Treatment Start (Week) | Treatment End (Week) |
|  | Control (Non-diabetic) | Standard pellet diet (SPD) | - | 12 | - | 21 |
|  | Diabetic control | SPD + 0.1% DMSO (oral) | 4 | | | |
|  | DN control | SPD +0.1% DMSO (oral) | - | 12 | - | 21 |
|  | Vitamin D | 5000 IU/kg b.w. weekly thrice for 9 weeks (oral) | 4 | 12 | 13 | 21 |
|  | Vitamin D | 8000 IU/kg b.w. weekly thrice for 9 weeks (oral) | 4 | 12 | 13 | 21 |
|  | Vitamin D + Metformin | Vitamin D: 5000 IU/kg b.w. + Metformin: 250 mg/kg b.w. weekly thrice for 9 weeks (oral) | 4 | 12 | 13 | 21 |
|  | Vitamin D + Metformin | Vitamin D: 8000 IU/kg b.w. + Metformin: 250 mg/kg b.w. weekly thrice for 9 weeks (oral) | 4 | 12 | 13 | 21 |
|  | Metformin | 250mg/kg b.w. weekly thrice for 9 weeks (oral) | 4 | 12 | 13 | 21 |

**Note:** Strategy 1 evaluates prophylactic (early treatment). Strategy 2 evaluates therapeutic (post-DN confirmation) intervention. All treatments were administered orally, three times per week. DN confirmation was based on microalbuminuria (>30mg/24hr) at week 12.


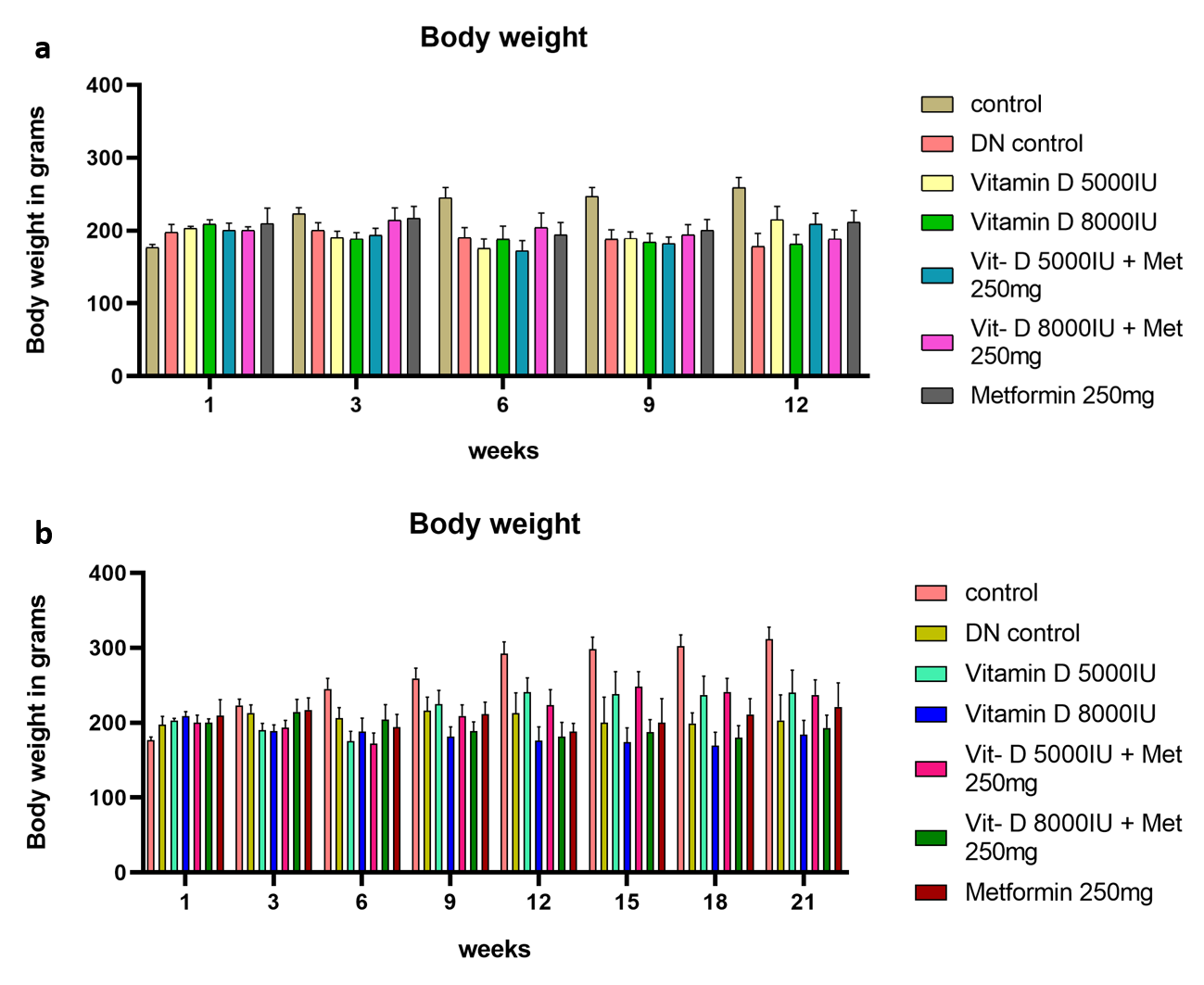


Figure S1a: Effect of vitamin D and metformin on body weight of STZ-induced diabetic rats during the 12-week experimental period (Strategy 1). Figure S1b: Effect of vitamin D and metformin on body weight of STZ induced DN rats during the 21-week experimental period (Strategy2).


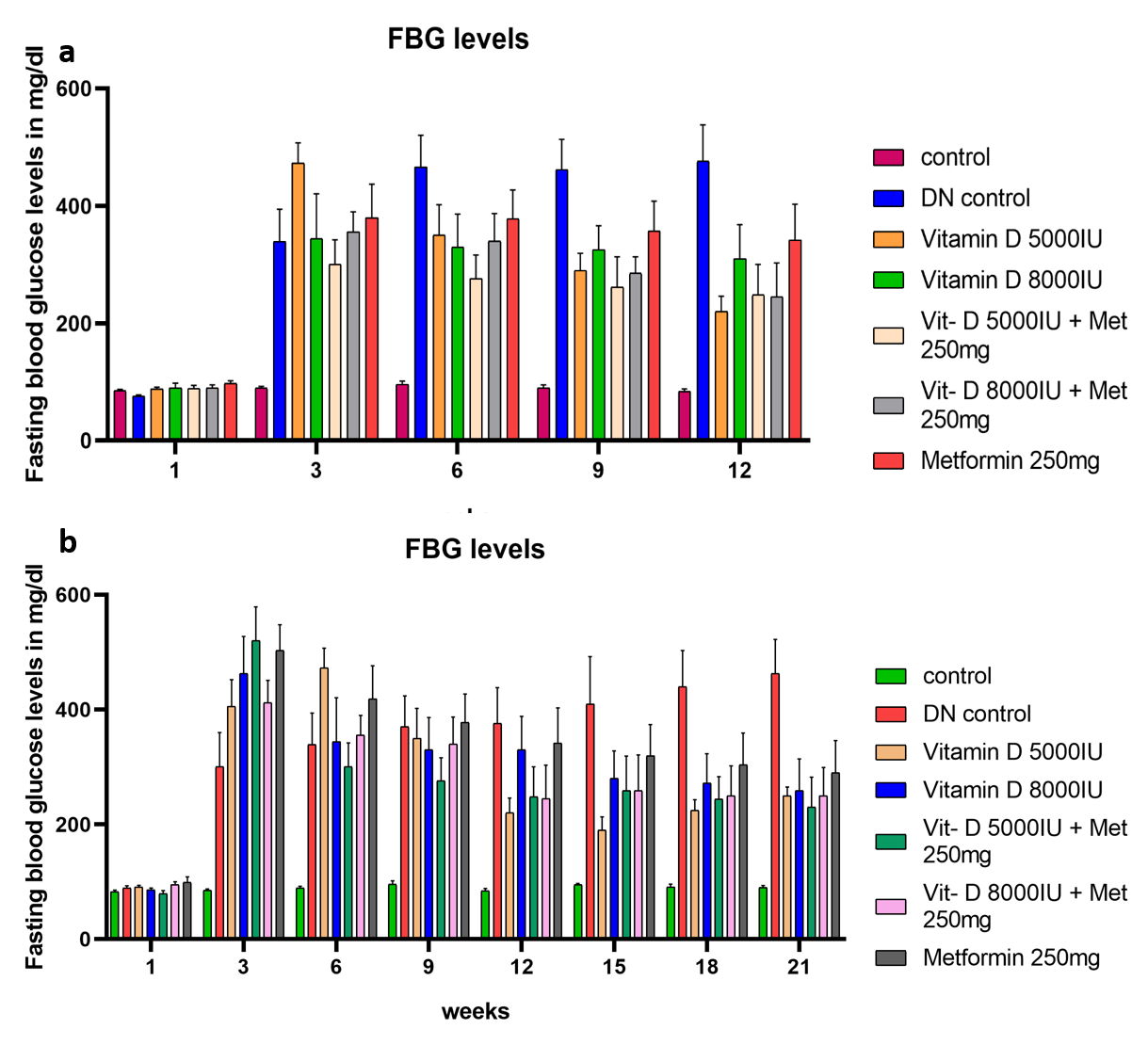


Figure S2a: Effect of vitamin D and metformin on FBG levels in STZ-induced diabetic rats during the 12-week experimental period (Strategy 1). Figure S2b: Effect of vitamin D and metformin on FBG levels in STZ-induced DN rats during the 21-week experimental period (Strategy2).

Table S2: Effect of vitamin D and Metformin on serum markers in STZ induced diabetic rats.

|  | Control | Diabetic control | DN  Control | Vitamin D-5000IU | Vitamin D-8000IU | Vitamin D+Metformin (5000IU+200mg) | Vitamin D+Metformin (8000IU+250mg) | Metformin (250mg) |
| --- | --- | --- | --- | --- | --- | --- | --- | --- |
| **Lipid profile** | | | | | | | | |
| Cholesterol (mg/dL) | 39.5±0.5 | 70±1.15 | 45.4±1.7^**,##^ | 42.7±2.1^***,###^ | 41.4±5.7^***,###^ | 49.6±5.4^#^ | 35.14±3.9^****,####^ | 48±3.14*,# |
| Triglycerides (mg/dL) | 79.5±2.5 | 168±4.4^**^ | 153±3.9 | 100±10.2 | 93±7.5^###^ | 90.4±9.3 | 83±6.4^#^ | 104±8.5 |
| HDL (mg/dL) | 27±1 | 32.25±1.5 | 28.6±1.03 | 36.6±3.6 | 30.6±2 | 30.5±2.9 | 26±8.4 | 25±1.6 |
| LDL (mg/dL) | 26.6±1.4 | 4.65±0.98 | -9.8±1.5 | 172±34 ^#,++^ | 178±58 ^#,++^ | 168±25 ^+^ | 99.76±17 | 173.7±6.2^#,+^ |
| VLDL (mg/dL) | 15.9±1.9 | 33.6±0.89^**^ | 26.6±1.48 | 26.5±2.25 | 18.74±1.5 ^##^ | 23.48±3.8 | 22.72±1.8 ^#^ | 24.84±2.3 |
| **Renal function markers** | | | | | | | | |
| Phosphorus (mg/dL) | 6.25±0.25 | 3.45±0.2 ^**^ | 6±0.33 ^##^ | 4.45±0.38 | 4.6±0.5 | 2.15±0.7 ^***,+++^ | 4.19±0.5 | 4.9±0.9 |
| Calcium (mg/dL) | 11.78±0.2 | 11.3±0.4 | 11.5±0.2 | 13±0.87 | 12.7±1.9 | 12.16±1.9 | 11.43±1.2 | 11.6±0.4 |
| Urea (mg/dL) | 42.5±0.5 | 38.5±0.5 | 41.2±0.9 | 50.6±1.7 | 45.8±2.5 | 47.86±4.8 | 43.57±2.6 | 35.25±4.5 |
| Uric acid (mg/dL) | 0.69±0.01 | 0.97±0.03 | 2.1±0.06 | 0.56±0.08 ^++^ | 0.51±0.16 ^++^ | 0.49±0.09 ^#,++^ | 0.35±0.06 ^##,+++^ | 0.5±0.13 ^+^ |
| Creatinine (mg/dL) | 0.69±0.01 | 0.64±0.02 | 0.6±0.05 | 0.09±0.008 ^****, ####, ++++^ | 0.06±0.02 ^****, ####, ++++^ | 0.15±0.017 ^***,####, +++^ | 0.15±0.05 ^***,####, ++++^ | 0.15±0.14 ^*, ##, +^ |
| BUN (mg/dL) | 19.8±0.23 | 17.9±0.26 | 19.3±0.45 | 36.01±6.65 | 47.28±18.7 | 19.19±27 | 22.07±24 | 36.31±19 |
|  | | | | | | | | |
| Vitamin D (ng/ml) | 38±13.48 | 13.1±0.17 | 10.75±2.3 | 63.3±10.47 ^#, +^ | 69.67±15 *, ^#, +^ | 68.29±23 ***, ^##, ++^ | 76.19±8.4 ^***, ##, +++^ | 86.54±13.6 ^****, ###, +++^ |
| **Oxidative Stress Markers** | | | | | | | | |
| Hydro peroxides (nmol HP/mg protein) | 0.25±0.3 | 0.35±0.1 ^***^ | 0.42±0.14 ^***^ | 0.38±0.16 ^****, ##^ | 0.37±0.15 ^****^ | 0.33±0.07 ^**, +++^ | 0.28±0.08 #, ^++++^ | 0.29±0.3 ^++++^ |
| Nitrite levels (µmol/L) | 0.52±0.1 | 0.64±0.3 ^****^ | 0.83±0.02 ^****, ####^ | 0.79±0.4 ^****, ####^ | 0.78±0.9 ^****, ####^ | 0.64±0.4 ^****, ++++^ | 0.59±0.3 ^*, ++++^ | 0.58±0.7 ^*,# ++++^ |
| **Antioxidant Defences** | | | | | | | | |
| Glutathione Reductase (U/mg protein) | 0.035±0.01 | 0.03±0.5 ^*^ | 0.02±0.15 ^****, ###^ | 0.032±0.1 ^++++^ | 0.034±0.08 ^++++^ | 0.053±0.8 ^****, ####, ++++^ | 0.07±0.8 ^****, ####, ++++^ | 0.03±0.5 ^++++^ |
| Superoxide Dismutase (U/ml) | 75.6±0.17 | 73.9±0.06 ^***^ | 72.75±0.15 ^****, ##^ | 75.81±0.1 ^####, ++++^ | 76±0.5 ^####, ++++^ | 76.4±0.3 ^**, ####, ++++^ | 81±0.15 ^****, ####, ++++^ | 77±0.9 ^****,####, ++++^ |
| Glutathione peroxidase (U/ml) | 44.46±0.6 | 30.4±0.13 ^****^ | 26.47±0.61 ^****, ####^ | 36.91±0.58 ^****, ####, ++++^ | 38.58±0.66 ^****, ####, ++++^ | 47.31±0.1 ^****, ####, ++++^ | 49.49±0.25 ^****, ####, ++++^ | 46.2±1.25 ^****,####, ++++^ |

Values are represented as mean ± SE. One-way ANOVA followed by Tukey’s post-hoc test was used for multiple group comparisons. Superscripts *, **, *** & **** indicate significance at p-value <0.05, <0.01, <0.001 &, 0.0001 respectively, with all groups compared to the control group. Superscripts #, ##, ###, & #### indicate significance at p-value <0.05, <0.01, <0.001 & <0.0001 respectively, with all groups compared to diabetic control group. Superscripts +,++,+++, & ++++ indicate significance at p-value <0.05, <0.01, <0.001 & <0.0001 respectively, with all groups compared to DN control group.

Table S3: Effect of vitamin D and Metformin on serum markers in STZ induced DN rats.

|  | Control | Diabetic control | DN Control | Vitamin D-5000IU | Vitamin D-8000IU | Vitamin D+Metformin (5000IU+200mg) | Vitamin D+Metformin (8000IU+250mg) | Metformin (250mg) |
| --- | --- | --- | --- | --- | --- | --- | --- | --- |
| **Lipid profile** | | | | | | | | |
| Cholesterol (mg/dL) | 56.8±1.5 | 73.8±2.2 | 50.3 ±4.6 | 42.47±1 | 47.59±1.9 | 45.07±2.19 | 58±3.22 | 46.48±1.8 |
| Triglycerides (mg/dL) | 167.5±2.5 | 164.3±3.6 | 172±3.9 | 109.7±3.3 **^#^** | 88.65±3.7 ^*,###, +^ | 99.6±4 ^##^ | 92.8±4.2 ^#^ | 142±2.6 |
| HDL (mg/dL) | 28.5±0.53 | 37.9±1.36 | 26.7±1.21 | 29.9±0.76 | 26.2±1.78 | 23.5±3.76 | 29.2±1.17 | 28.3±3.6 |
| LDL (mg/dL) | -5.1±1.48 | 3.06±3.72 | -11±5 | -9±1.67 | 3.65±2.83 | 1.61±1.82 | 10.3±3.8 ^++^ | -10±1.82 |
| VLDL  (mg/dL) | 33.5±0.5 | 32.9±0.73 | 34.5±0.79 | 21.9±0.66^****,####,++++^ | 17.7±0.75^****,####,++++^ | 19.9±0.81^****,####,++++^ | 18.6±0.85^****,####,++++^ | 28.4±0.53^+^ |
| **Renal function markers** | | | | | | | | |
| Phosphorus (mg/dL) | 7±0.27 | 8.7±0.4 | 6.3±0.36 ^#^ | 5.7±0.12 ^##^ | 5.16±0.43 ^###^ | 5.28±0.49 ^##^ | 5.65±0.41 ^##^ | 7.1±0.5 |
| Calcium (mg/dL) | 10.9±.03 | 11±0.13 | 10.7±0.32 | 10.5±0.1 | 9.86±0.35 | 9.52±0.17 | 10.2±0.29 | 10.8±0.21 |
| Urea (mg/dL) | 44.3±0.52 | 71.7±2.27 | 80.9±2.01 | 36.1±1.21 | 50.9±4.49 | 37.5±3.5 | 51.7±4.45 | 47.2±1.18 |
| Uric acid (mg/dL) | 0.83±0.06 | 1.12±.022 | 2.03±0.12 | 1.37±0.09 | 1.5±0.26 | 1.32±0.25 | 1.61±.056 | 0.95±0.2 |
| Creatinine (mg/dL) | 0.72±0.07 | 0.67±0.01 | 1±0.01 | 0.64±.001 | 0.66±.016 | 0.71±0.02 | 0.78±0.06 | 0.78±0.02 |
| BUN (mg/dL) | 0.33±0.04 | 0.32±0.01 | 0.32±0.01 | 0.36±0.01 | 0.31±0.01 | 0.33±0.01 | 0.36±0.03 | 0.37±0.01 |
| **Vitamin D levels** | | | | | | | | |
| Vitamin D (ng/mL) | 37.8±13.5 | 12.8±2.89 ^****^ | 10.1±0.26 ^****,####^ | 76.9±5.8 ^****, ####, ++++^ | 63.3±8.34 ^****, ####, ++++^ | 82.2±9.15 ^****, ####, ++++^ | 62.4±12.1 ^****, ####, ++++^ | 34.3±12 ^####, ++++^ |
| **Oxidative Stress Markers** | | | | | | | | |
| Hydroperoxides (nmol HP/mg protein) | 0.31±0.05 | 0.34±0.06 ^**^ | 0.38±0.03 | 0.35±0.01 | 0.31±0.08 ^++^ | 0.31±0.05 ++ | 0.24±0.02 ^*, ###^ | 0.29±0.03 ^+++^ |
| Nitrite levels (µmol/L) | 0.71±0.05 | 0.85±0.01 ^****^ | 0.91±0.05 ^****^ | 0.85±0.02 ^****, +^ | 0.87±0.01 ^****^ | 0.86±0.06 ^****^ | 0.78±0.006 ^**, ##, ++++^ | 0.84±0.02 ^****, +^ |
| **Antioxidant Defences** | | | | | | | | |
| Glutathione Reductase (U/mg protein) | 0.032±0.1 | 0.03±0.3 | 0.011±0.01^****, ####^ | 0.012±0.01^****, ####^ | 0.013±0.01^****, ####^ | 0.011±0.01^****, ####^ | 0.034±0.12 ^#, ++++^ | 0.03±0.3 ^++++^ |
| Superoxide Dismutase (U/ml) | 77.1±0.05 | 74.1±0.02 ^****^ | 72.1±0.05 ^****, ####^ | 75.4±0.02 ^****, ####, ++++^ | 75.6±0.02 ^****, ####, ++++^ | 75.8±0.02 ^****, ####, ++++^ | 77.4±0.01 ^****, ####, ++++^ | 76.3±0.03 ^****, ####, ++++^ |
| Glutathione peroxidase (U/ml) | 46.03±0.09 | 40±0.13 ^****^ | 35.5±0.15^****, ####^ | 47.45±0.2 ^****, ####, ++++^ | 45.93±0.06 ^####, ++++^ | 47.41±0.2 ^***, ####, ++++^ | 49.8±0.19 ^****, ####, ++++^ | 48.9±0.05 ^****, ####, ++++^ |

Values are represented as mean ± SE. One-way ANOVA followed by Tukey’s post-hoc test was used for multiple group comparisons. Superscripts *, **, *** & **** indicate significance at p-value <0.05, <0.01, <0.001 &, 0.0001 respectively, with all groups compared to the control group. Superscripts #, ##, ###, & #### indicate significance at p-value <0.05, <0.01, <0.001 & <0.0001 respectively, with all groups compared to diabetic control group. Superscripts +,++,+++, & ++++ indicate significance at p-value <0.05, <0.01, <0.001 & <0.0001 respectively, with all groups compared to DN control group.
